# Viral manipulation of STAT3: Evade, exploit, and injure

**DOI:** 10.1371/journal.ppat.1006839

**Published:** 2018-03-15

**Authors:** Armando Andres Roca Suarez, Nicolaas Van Renne, Thomas F. Baumert, Joachim Lupberger

**Affiliations:** 1 Inserm, U1110, Institut de Recherche sur les Maladies Virales et Hépatiques, Strasbourg, France; 2 Université de Strasbourg, Strasbourg, France; 3 Pôle Hépato-digestif, Institut Hospitalo-universitaire, Hôpitaux Universitaires de Strasbourg, Strasbourg, France; University of Alberta, CANADA

## Abstract

Signal transducer and activator of transcription 3 (STAT3) is a key regulator of numerous physiological functions, including the immune response. As pathogens elicit an acute phase response with concerted activation of STAT3, they are confronted with two evolutionary options: either curtail it or employ it. This has important consequences for the host, since abnormal STAT3 function is associated with cancer development and other diseases. This review provides a comprehensive outline of how human viruses cope with STAT3-mediated inflammation and how this affects the host. Finally, we discuss STAT3 as a potential target for antiviral therapy.

## Signal transduction through the STAT3 pathway

### STAT3 is a transcription factor activated by tyrosine phosphorylation

**Fig 1:**
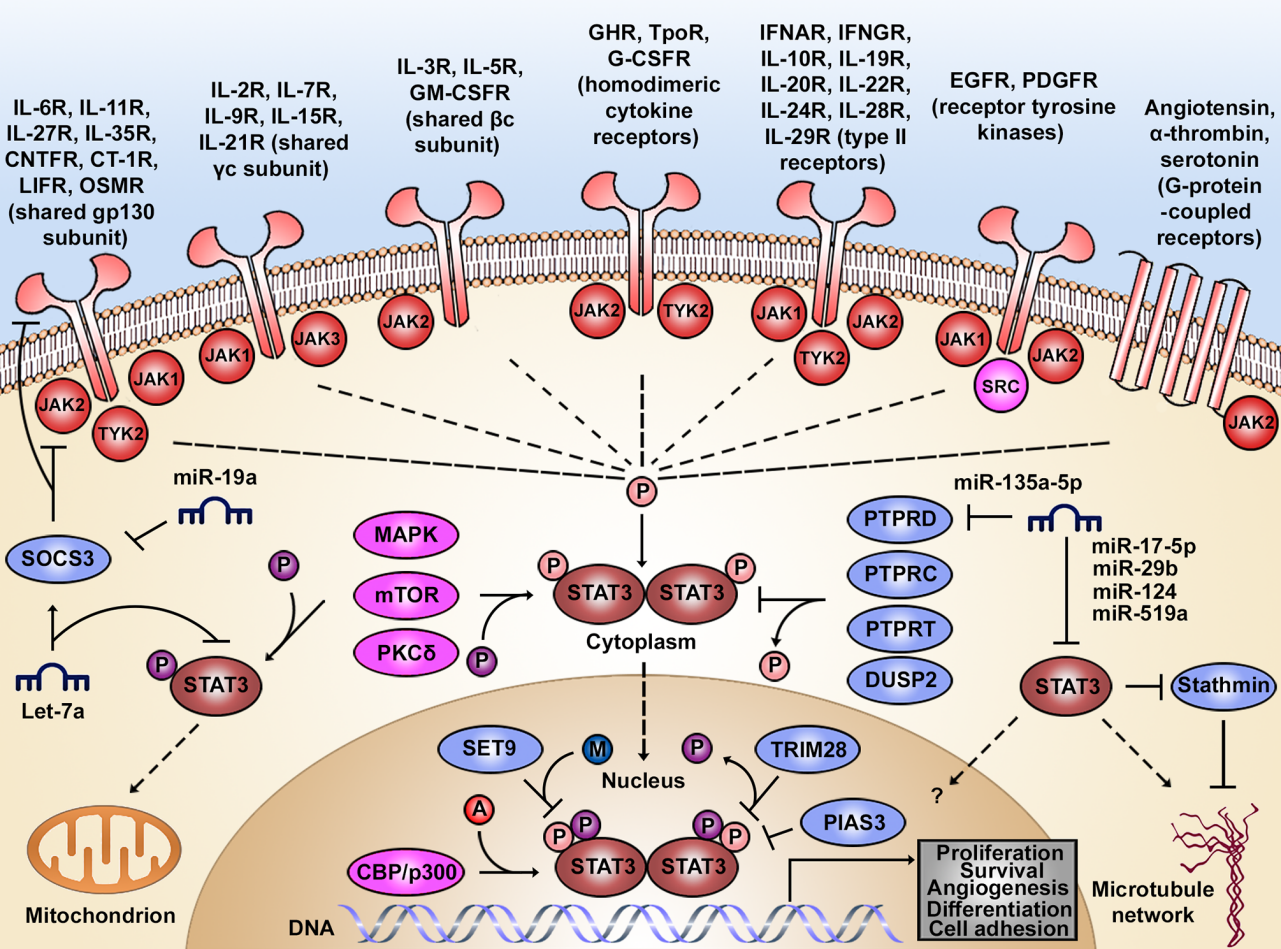
Regulatory circuits of the STAT3 signaling pathway. STAT3 can be activated by a wide range of ligands binding to cytokine, growth factor, or G-protein-coupled receptors. With the exception of receptor tyrosine kinases, these receptors lack intrinsic kinase activity and thus act by recruiting adaptor kinases (e.g., JAKs, SRC) to propagate downstream signals. As a result, STAT3 is phosphorylated at tyrosine 705 (pY705, pink), forms homodimers or heterodimers, and translocates to the nucleus, where it transcribes regulators of various cellular processes. Additionally, STAT3 can be phosphorylated at serine 727 (pS727, purple) by serine/threonine kinases (e.g., MAPK, mTOR, PKCδ), which enhance STAT3 transcriptional activity in the nucleus or direct STAT3 to mitochondria. Acetylation at lysine 685 (K685, red) by histone acetyltransferases (e.g., CREB binding protein CBP/histone acetyltransferase p300) or methylation at lysine 140 (K140, blue) by histone methyltransferases (e.g., SET9) favor or impair STAT3 transcriptional activity, respectively. Unphosphorylated STAT3 exhibits regulatory functions in the nucleus or can be retained in the cytoplasm, where it associates with microtubules and focal adhesions. The activity of STAT3 is tightly regulated by phosphatases (e.g., PTPRD), SOCS3, PIAS3, and miRNAs that fine-tune the temporal pattern of STAT3 activity and its other pathway components. All miRNAs are degrading the mRNAs of the indicated proteins. A, acetylation; CBP, CREB-binding protein; CT-1R, cardiotrophin 1 receptor; CNTFR, ciliary neurotrophic factor receptor; DUSP2, dual specificity protein phosphatase 2; EGFR, epidermal growth factor receptor; GHR, growth hormone receptor; G-CSFR, granulocyte colony-stimulating factor receptor; GM-CSFR, granulocyte-macrophage colony-stimulating factor receptor; gp130, glycoprotein 130; IFNAR, interferon alpha receptor; IFNGR, interferon gamma receptor; IL, interleukin; JAK, Janus kinase; K140, lysine 140; K685, lysine 685; LIFR, leukemia inhibitory factor receptor; MAPK, mitogen-activated protein kinase; M, methylation; miRNA, microRNA; mTOR, mechanistic target of rapamycin; OSMR, oncostatin-M-specific receptor; P, phosphorylation; p300, histone acetyltransferase p300; PDGFR, platelet-derived growth factor receptor; PIAS3, protein inhibitor of activated STAT protein 3; PKCδ, protein kinase C delta type; pS727, phospho-serine 727; PTPRC, receptor-type tyrosine-protein phosphatase C; PTPRD, receptor-type tyrosine-protein phosphatase D; PTPRT, receptor-type tyrosine-protein phosphatase T; pY705, phospho-tyrosine 705; SET9, histone-lysine N-methyltransferase SET9; SOCS3, suppressor of cytokine signaling 3; SRC, proto-oncogene tyrosine-protein kinase; STAT3, signal transducer and activator of transcription 3; TpoR, thrombopoietin receptor; TRIM28, tripartite motif-containing protein 28.

Signal transducer and activator of transcription 3 (STAT3) was first described in 1994 as a central transcription factor in acute inflammation [1]. Since then, STAT3 has been shown to regulate a wide spectrum of biological programs, including inflammation, tissue regeneration, cell proliferation, cell survival, cellular differentiation, angiogenesis, chemotaxis, and cell adhesion. This functional pleiotropy can be partially explained by the broad number of ligands that lead to STAT3 activation after binding to their respective cytokine receptors [2]. Upon cytokine binding, there is typically recruitment and reciprocal trans-phosphorylation of tyrosine kinases of the Janus kinase (JAK) family comprising JAK1, JAK2, JAK3, and tyrosine kinase 2 (TYK2) [3,4,5]. They, in turn, recruit and phosphorylate STAT3 (p-STAT3) at the highly conserved tyrosine residue 705 (pY705) [6], resulting in the formation of STAT3 homo- or heterodimers with signal transducer and activator of transcription 1 (STAT1) or signal transducer and activator of transcription 5 (STAT5) [7]. Subsequently, the activated signal transducer and activator of transcription (STAT) dimers translocate to the nucleus and facilitate gene transcription after binding to genomic DNA. Many pathways thus converge in STAT3-mediated gene-expression (Fig 1).

### Regulation of STAT3 activation

STAT3 activity is additionally regulated by several post-translational modifications. First, phosphorylation at serine 727 (pS727) by a variety of serine/threonine kinases, such as the mitogen-activated protein (MAP) kinases, mechanistic target of rapamycin (mTOR), and protein kinase C delta type (PKCδ), increases transcriptional activity even further [8]. In mitochondria, pS727 promotes cellular respiration independently from pY705 [9]. Second, STAT3 can be reversible acetylated on K685 by histone acetyltransferase CBP/p300, prolonging transcriptional activity [10]. Contrarily, K140 methylation by histone methyltransferase SET9 impairs transcription [11].

Additional negative feedback regulators include the protein phosphatases receptor-type tyrosine-protein phosphatase C (PTPRC), receptor-type tyrosine-protein phosphatase D (PTPRD), receptor-type tyrosine-protein phosphatase T (PTPRT), and dual specificity protein phosphatase 2 (DUSP2) that hydrolyze p-STAT3 or upstream pathway members [12, 13, 14, 15]. Suppressor of cytokine signaling 3 (SOCS3) prevents STAT3 activation by shielding phospho-tyrosine residues of upstream kinases [16,17], while protein inhibitor of activated STAT protein 3 (PIAS3) prevents binding of STAT3 dimers to DNA [18]. In the nucleus, the phosphorylation and transcriptional activity of STAT3 pS727 is negatively regulated by tripartite motif-containing protein 28 (TRIM28), which binds directly to the central coiled-coil and DNA-binding domains of STAT3 [19]. Furthermore, several microRNAs (miRNAs) directly target *STAT3* mRNA, including Let-7a [20], miR-17-5p [21], miR-29b [22], miR-124 [23], and miR-519a [24]. Let-7a also exerts an indirect effect on STAT3 by promoting SOCS3 expression [25]. STAT3-activating miRNAs include miR-24 and miR-629 that impair miR-124 expression via *HNF4A* mRNA silencing [26]. Similarly, miR-135a-5p and miR-19a enhance pY705 phosphorylation by respectively targeting the mRNA of *PTPRD* and *SOCS3* [27,28].

Although STAT3 phosphorylation is often considered a prerequisite for its transcriptional activity, unphosphorylated STAT3 (u-STAT3) can promote the expression of genes related to cell cycle progression [29,30]. Finally, cytoplasmic STAT3 promotes cell migration by interacting with stathmin, a microtubule destabilizer [31].

## Physiological role of STAT3 in inflammation

In mammalian organisms, tissue injuries inflicted by pathogens are met by the release of inflammatory mediators and local infiltration of white blood cells. This eliminates foreign material, removes damaged tissue components, and clears the way for repair. STAT3 plays an essential role in these processes by enabling the expression of a variety of genes in response to specific external signals sensed by cell-surface receptors [32]. Not all cell types and tissues have the same expression patterns of these receptors and their signaling cascade mediators. Therefore, the functional consequence of STAT3 activation is highly context-dependent, which can often lead to conflicting information. As illustrated in the following examples, this is particularly true for the role of STAT3 in inflammation, since it is either able to promote or suppress this process.

### IL-6/STAT3 pathway promotes inflammation

Interleukin 6 (IL-6) is a classic proinflammatory cytokine that signals through STAT3 as part of the acute phase response (APR), a nonspecific reaction of the innate immune system to pathogen infection. During acute inflammation, IL-6 is produced in the lesion site to attract neutrophils and increase granulopoiesis [33]. Upon extravasation at the site of injury, neutrophils produce soluble interleukin 6 receptor alpha (sIL-6Rα), which in complex with IL-6 binds to glycoprotein 130 (gp130) at the membrane of resident tissue cells. This process is known as the *trans*-signaling pathway [34], which subsequently leads to a switch in chemokine expression attracting monocytic and T cells [35,36]. Upon the arrival of monocytic cells in the inflammation site, IL-6 signals govern their transformation into macrophages [37]. Pathogens are thus initially confronted in their initial microenvironment with a potent IL-6 stimulus, which is mounted by the host to combat their very presence.

Apart from the lesion site, the IL-6/STAT3 proinflammatory signaling axis functions in many other cellular and tissue compartments. In secondary lymphoid tissues, where the adaptive immune response takes place, IL-6-mediated STAT3 activation promotes the proliferation and survival of T and B cell populations [38,39]. In addition, together with transforming growth factor beta (TGF-β), the IL-6/STAT3 axis is crucial for differentiating naive CD4^+^ T cells into Th17 cells [40,41], limiting the generation of regulatory CD4^+^ T cells (T_reg_ cells) [42]. Moreover, IL-6 promotes the differentiation of follicular helper T cells (T_FH_ cells) via STAT3 [43,44], effectively linking together T and B cell responses [45].

### IL-10/STAT3 pathway suppresses inflammation

Interleukin 10 (IL-10) also activates STAT3, but unlike IL-6 the IL-10/STAT3 axis has powerful anti-inflammatory properties. Its function is essential to restrain unwanted immune responses and prevent autoimmune pathologies [46]. IL-10 only exerts an effect on immune cells, as they are the only cells to have the interleukin 10 receptor alpha (IL-10RA). This IL-10 receptor is highly expressed in monocytic cells and macrophages but also to a lesser extent in NK cells, CD4^+^ and CD8^+^ T cells, B cells, dendritic cells (DCs), and mast cells [47]. Until recently it was unclear how, in cells responsive to both IL-6 and IL-10, STAT3 orchestrates such opposing functions. In fact, SOCS3 is critical for selecting the transcriptional response. While IL-6 signaling is selectively inhibited by SOCS3 binding to gp130, SOCS3 does not interfere with IL-10R-mediated STAT3 activation [48]. As an effect, STAT3 activation is transient and proinflammatory in response to IL-6, while long lasting and anti-inflammatory in IL-10 [49].

IL-10 exerts its anti-inflammatory effect by suppressing T helper 1 (T_H_1) cell responses [50] and regulating apoptosis in B cells [51]. In addition, IL-10/STAT3 is necessary for generation of tolerogenic DCs and of induced T_reg_s out of naïve CD4^+^ T cells [52].

### Interferon activation of STAT3

Upon viral infection, type I and type II interferons (IFNs) initiate a canonical antiviral transcriptional program through STAT1 and STAT2, which results in an inflammatory, proapoptotic, and antiproliferative state [53]. At the same time, IFNs induce STAT3 activation [54,55], which provides a negative feedback by favoring cell proliferation and survival and thus resulting in gene expression with anti-inflammatory properties [56]. In support of this model, silencing of STAT1 or STAT3 expression by RNA interference confirmed the role of STATs as important determinants of IFN-α receptor (IFNAR) function [57] and emphasizes the role of STAT3 to restrain STAT1-mediated proinflammatory signaling [58].

In this context, an initial proinflammatory response to IFNs is mediated by STAT1, which expression is far more abundant, while STAT3-mediated gene induction is prevented by the SIN3 transcription regulator family member A complex (SIN3A). This multimolecular complex, containing histone deacetylases 1 (HDAC1) and 2 (HDAC2), inactivates STAT3 by deacetylation [59]. It has been suggested that only in a second phase is STAT3 activity increased, leading to a sequential counterbalance to the initial flare of apoptosis and decrease in proliferation mediated by IFNs [60].

A potential regulatory layer that remains poorly understood is the role of STAT1 and STAT3 heterodimers induced by IFNs. On one hand, STAT1 and STAT3 heterodimers have been described to bind regulatory elements present in promoters of interferon-stimulated genes (ISGs) such as γ-activated sequence (GAS), supporting a potential antiviral role of STAT1 and STAT3 heterodimers [61]. On the other hand, it has been proposed that STAT1 and STAT3 heterodimers can effectively quench STAT1 and thus provide negative feedback in a later phase of the IFN response [57]. Whatever the effect of STAT1 and STAT3 heterodimers on viral infection, either proviral or antiviral, it provides another layer of potential manipulation for viral gene products that warrants further research.

The suggested temporal dynamics of STAT biology may explain the serious consequences of persistent viral infections, as in the case of hepatitis C virus (HCV) [60]. Here, sustained type I and II IFN signaling may drastically alter the initial STAT dimerization balance, enabling a more pronounced proliferative role of STAT3 and hence increasing oncogenic pressure on hepatocytes.

## Role of STAT3 in regeneration and disease

Upon infection, inflammatory cytokines trigger cell signaling in local stem cells or differentiated cells. Among other transcription factors, this eventually leads to the activation of STAT3 that mediates regenerative gene-expression programs. These genes include growth factors, cell-cycle stimulators, cell death inhibitors, and genes promoting dedifferentiation and cell motility and migration [62]. The task of STAT3 in regenerative inflammation is well studied in the liver, a model for organ regeneration as it can easily restore functional capacity after partial resection through compensatory hyperplasia [63,64]. In the liver, the inflammatory response following injury instigates the regenerative process [65]. As part of the APR, liver-residing macrophages (Kupffer cells) release proinflammatory cytokines such as IL-6 and tumor necrosis factor alpha (TNF-α) [66]. These inflammatory cytokines are important components of priming pathways that help sensitize hepatocytes to proliferative signals, such as hepatocyte growth factor (HGF) and epidermal growth factor (EGF) [67]. However, when liver injury persists, as in the case of chronic viral hepatitis, liver inflammation paired with constant STAT3 activity fosters the development of hepatocellular carcinoma (HCC) [27]. A similar oncogenic role of STAT3 has been observed in a wide variety of other malignancies such as colorectal, lung, prostate, gastric, and breast cancers [68].

Given the extensive role of STAT3 in many physiological processes, it is only logical that its perturbation entails a wide variety of pathological consequences. This is exemplified by loss-of-function mutations in the STAT3 gene that lead to the autosomal dominant hyper-immunoglobulin E (IgE) syndrome (AD-HIES) [69]. These patients exhibit an immunodeficiency complex that presents with recurrent episodes of pneumonia and other lung abnormalities, abnormally high levels of IgE, eosinophilia, eczema, and skeletal and connective tissue abnormalities. Inadequate inflammatory capacity due to a broken IL-6/STAT3 axis curtails the APR and leads to "cold" skin abscesses (i.e., without inflammatory signs). As STAT3 is necessary for generating Th17 cells, a defective Th17 response and increased susceptibility for microbial infections are hallmarks of AD-HIES. On the other hand, the defects in the anti-inflammatory IL-10/STAT3 pathway lead to reduced peripheral tolerance, which is clinically translated in atopic dermatitis. Finally, AD-HIES patients exhibit a marked reduction in memory T cells and increased latency of herpesviruses such as varicella-zoster virus (VZV) and Epstein–Barr virus (EBV) [70].

## Molecular mechanisms of viral STAT3 manipulation

### Viral stimulation of STAT3 function

**Fig 2:**
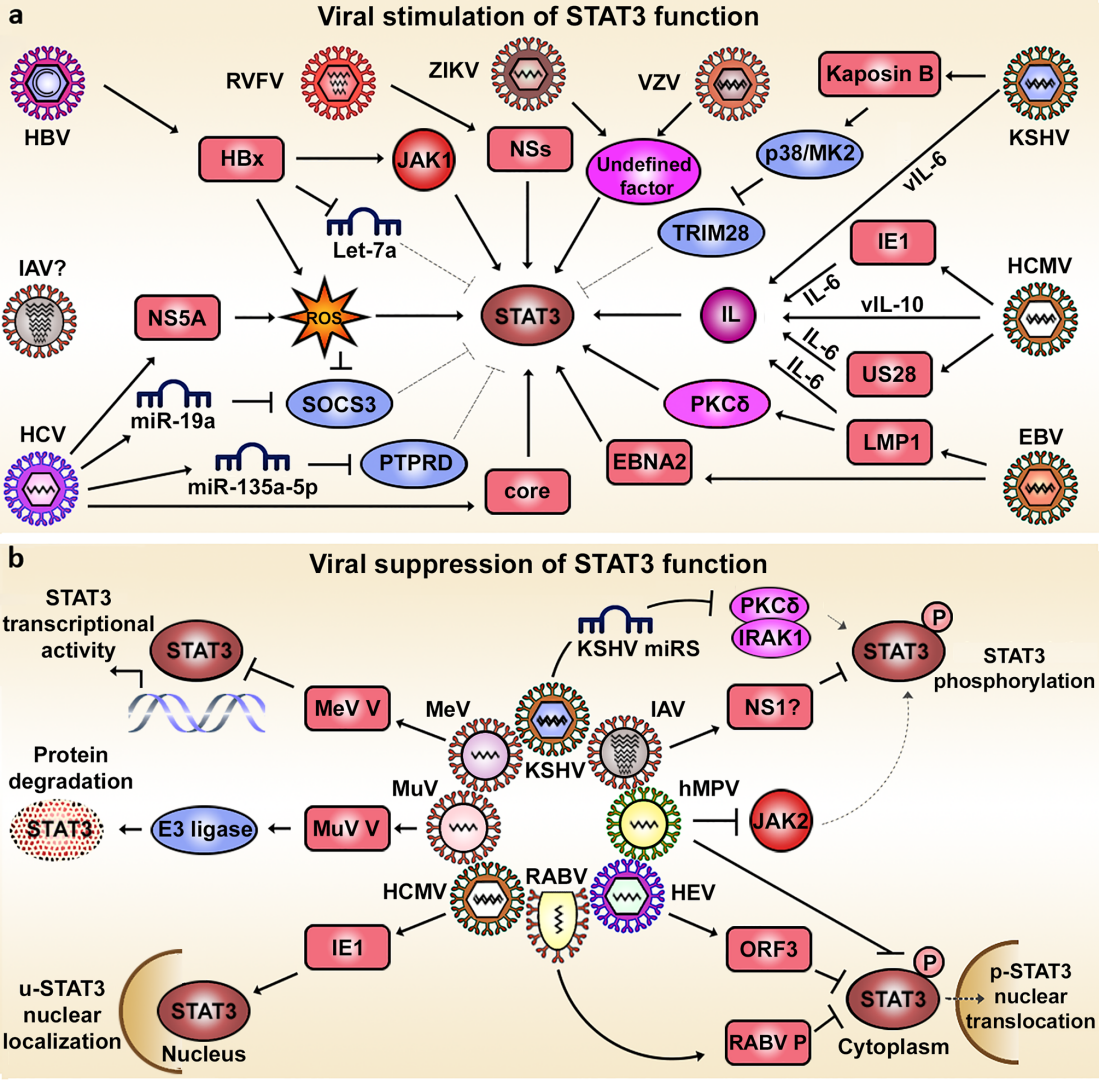
Viral manipulation of the STAT3 signaling pathway. **(A)** Viruses activating STAT3 function and the mechanisms involved. Viral proteins such as HBx, NS5A, core, NSs, EBNA2, LMP1, US28, and IE1 induce STAT3 activation either directly or by favoring the action of upstream positive regulators. Viruses like HCMV and KSHV code for homologues of human interleukins such as IL-10 and IL-6. Alternatively, virus-induced activation of STAT3 can be achieved by the inhibition of negative regulators such as SOCS3, PTPRD, TRIM28, and Let-7a. In the case of some viruses, STAT3 activation (VZV and ZIKV) or STAT3-mediated effects (IAV) have been described, but the mechanisms involved have not been fully elucidated. All miRNAs are degrading the mRNAs of the indicated proteins. **(B)** Viruses suppressing STAT3 function and the mechanisms involved. Virus-mediated inactivation of STAT3 can be attained by decreasing its phosphorylation (KSHV, IAV, and hMPV), inducing STAT3 protein degradation (MuV), hampering its transcriptional activity (MeV), or altering its subcellular localization (HCMV, RABV, HEV, and hMPV). EBNA2, Epstein–Barr virus nuclear antigen 2; EBV, Epstein–Barr virus; HBV, hepatitis B virus; HBx, hepatitis B virus X protein; HCMV, human cytomegalovirus; HCV, hepatitis C virus; HEV, hepatitis E virus; hMPV, human metapneumovirus; IAV, influenza A virus; IE1, intermediate-early protein 1; IL-6, interleukin 6; IL-10, interleukin 10; IRAK1, interleukin 1 receptor-associated kinase 1; JAK1, Janus kinase 1; KSHV, Kaposi’s sarcoma-associated herpesvirus; LMP1, latent membrane protein 1; miRNA, microRNA; MeV, measles virus; MK2, mitogen-activated protein kinase 2; MuV, mumps virus; NS5A, non-structural protein 5A; NSs, non-structural proteins; P, phosphorylation; PKCδ, protein kinase C delta type; PTPRD, receptor-type tyrosine-protein phosphatase D; RABV, rabies virus; ROS, reactive oxygen species; RVFV, Rift Valley fever virus; SOCS3, suppressor of cytokine signaling 3; STAT3, signal transducer and activator of transcription 3; TRIM28, tripartite motif-containing protein 28; u-STAT3, unphosphorylated STAT3; vIL-10, viral IL-10; vIL-6, viral IL-6; VZV, varicella-zoster virus; ZIKV, Zika virus.

As STAT3 activation is a pivotal event in the APR elicited by pathogen invasion, many viruses have evolved to thrive in a STAT3-driven microenvironment and have developed strategies to stimulate STAT3 signaling (Fig 2A, Table 1). For example, hepatitis B virus (HBV) promotes the formation of p-STAT3 dimers that bind specifically to an androgen-responsive element site present in the HBV enhancer 1 region and hence stimulates viral gene expression [71]. This is in part mediated by hepatitis B virus X protein (HBx), which induces pY705 phosphorylation via JAK1 [72] and down-regulates miRNA let-7a, a negative regulator of *STAT3* mRNA [20]. Additionally, HBV favors STAT3 activation by inducing reactive oxygen species (ROS), which results in epigenetic silencing of *SOCS3* mRNA via up-regulation of snail family transcriptional repressor 1 (SNAIL1) [73]. HCV requires STAT3 and therefore promotes STAT3 signaling to maintain infection [74]. HCV stimulates STAT3 directly by interaction with the HCV core protein [75] and indirectly through non-structural protein 5A (NS5A), which activates STAT3 via ROS induction [76]. Furthermore, miR-135a-5p is a negative regulator of STAT3 phosphatase PTPRD and is up-regulated in HCV-infected hepatocytes, leading to an enhanced STAT3 transcriptional activity [27]. Furthermore, HCV-infected hepatocytes secrete miR-19a within exosomes, down-regulating the expression of SOCS3 in hepatic stellate cells (HSCs) and promoting STAT3 phosphorylation [28]. Similarly, Rift Valley fever virus (RVFV) infection induces STAT3 (pY705) phosphorylation by the viral non-structural protein s (NSs) [77]. STAT3 activation is also a frequent feature of the Herpesviridae family. Human cytomegalovirus (HCMV) activates STAT3 through various mechanisms, depending on virus strain and cell type. In U373 MG astrocytes, viral protein US28 of the Titan strain induces IL-6 production, which in turn activates STAT3 in an auto- and paracrine fashion [78]. In hepatoma cells and primary human hepatocytes (PHHs), strains AD169 and HCMV-DB also activate STAT3 via IL-6 in an autocrine and/or paracrine mannerinfluenza strain H5N1 impairs pY705 phosphorylation at I87A [87]. Kaposi’s sarcoma-associated herpesvirus (KSHV) encodes a viral homologue of IL-6 (vIL-6) that signals through the same receptors as cellular IL-6 (IL-6Rα/gp130) but can also activate STAT3 in an IL-6Rα-independent manner in Hep3B liver cells [88]. In human endothelial cells, KSHV increases both pY705 and pS727 phosphorylation [19]. Though pY705 phosphorylation is transient, pS727 persists because the viral protein kaposin B activates the p38/MK2 pathway to suppress TRIM28, which is a negative regulator of pS727 phosphorylation [19]. STAT3 activation in DCs is believed to stem from virions interacting with dendritic cell-specific ICAM-3-grabbing nonintegrin 1 (DC-SIGN) at the cell’s surface, as antibody blockage of DC-SIGN reduces pY705 levels [89]. VZV induces pY705 phosphorylation in epidermal cells and T cells in vivo as well as in fibroblasts in vitro through unknown mechanisms [90]. Resveratrol, an inhibitor of kinases phosphorylating STAT3, hampers VZV infection, suggesting the involvement of host kinases [91]. Similarly, ZIKA virus (ZIKV) infection induces pY705 in primary retinal glial cells [92] and favors the activity of the IL-6/STAT3 pathway in blood mononuclear cells from infected rhesus monkeys, albeit without any known molecular mechanism [93].

**Table 1:** Virus/STAT3 interactions: Summary of observations and employed methods.

Virus	Observation	Method	Virus strain	Experimental system	Reference
**HBV**	Increased STAT3 (pY705) phosphorylation	In vitro viral protein expression (HBx)	adr4-derived sequence (genotype C)	Mouse hepatoma cell line (Hepa 1–6)	[72]
	Increased STAT3 protein and mRNA expression	In vitro viral protein expression (HBx)	adw-derived sequence (genotype A)	Human hepatoma cell lines (HepG2, SNU-182)	[20]
	Increased STAT3 (?) phosphorylation	HBV-expressing cells and patient-derived samples	ayw (genotype D)	Human hepatoma cell line (HepG2.2.15), HBV-positive HCC samples	[73]
**HCV**	Increased STAT3 (pY705) phosphorylation	In vitro HCV genomic replicon and virus infection	JFH-1 (genotype 2a)	Human hepatoma cell lines (Huh-7, NNeoC-5B)	[74]
	Increased STAT3 (pY705) phosphorylation	In vitro and in vivo viral protein expression (core)	Patient-derived sequence (genotype 1b)	Human hepatoma cell line (HepG2), Tg mice (C57BL/6)	[75]
	Increased STAT3 (pY705) phosphorylation	In vitro viral protein expression (NS5A)	Patient-derived sequence (genotype 1b)	Human hepatoma cell line (Huh-7)	[76]
	Up-regulation STAT3 responsive genes	In vitro HCV infection and patient-derived samples	Jc1 (genotype 2a chimera)	Human hepatoma cell line (Huh7.5.1) and HCV-positive HCC samples	[27]
	Increased STAT3 (?) phosphorylation	In vitro exposition to HCV-derived exosomes	JFH-1 (genotype 2a)	Primary HSCs	[28]
**RVFV**	Increased STAT3 (pY705) phosphorylation	In vitro viral protein expression (NSs) and RVFV infection	Recombinant MP12	Vero cells, HSAECs, and MEFs	[77]
**HCMV**	Increased STAT3 (pY705) phosphorylation	In vitro viral protein expression (US28) and HCMV infection	Titan	HEK293 and astrocytoma cell line (U373 MG)	[78]
	Increased STAT3 (?) phosphorylation	In vitro HCMV infection	HCMV-AD169, HCMV-DB	Human hepatoma cell line (HepG2) and PHHs	[79]
	Increased STAT3 (?) phosphorylation	In vitro vIL-10 stimulation		DCs	[81]
	Increased STAT3 (pY705/pS727) phosphorylation	In vitro vIL-10 stimulation		Primary human monocytic cells	[82]
	Increased u-STAT3 nuclear localization	In vitro viral protein expression (IE2) and HCMV infection	HCMV-AD169	Human embryonic lung fibroblasts (MRC-5) and astrocytoma cell line (U373)	[101]
**EBV**	Increased STAT3 (pY705) phosphorylation	In vitro viral protein expression (LMP1) and EBV infection	Recombinant EBV (Bx1)	HeLa cells, NPC cell line (CNE2)	[83]
	Increased STAT3 (pY705/pS727) phosphorylation	In vitro viral protein expression (LMP1)		Cervical carcinoma cell line (C33A)	[84]
	Increased STAT3 DNA-binding and transcriptional activity	In vitro viral protein expression (EBNA2)		HeLa, HEK293, and human Burkitt’s lymphoma B cell line (DG75)	[85]
**KSHV**	Increased STAT3 (pY705) phosphorylation	In vitro viral protein expression or stimulation (vIL-6)		Human hepatoma cell line (Hep3B)	[88]
	Increased STAT3 (pY705/pS727) phosphorylation	In vitro KSHV infection	BCBL-1-cell line-derived	HUVECs	[19]
	Increased STAT3 (pY705) phosphorylation	In vitro KSHV infection	BC3-cell line-derived	DCs	[89]
	Decreased STAT3 (pY705) phosphorylation	In vitro viral miRNAs expression	BCBL-1-cell line-derived	HUVECs	[102]
**VZV**	Increased STAT3 (pY705) phosphorylation	In vitro and in vivo VZV infection	Recombinant VZV (ORF10-GFP)	HELFs, primary tonsil T cells and human skin xenografts (mouse)	[90]
**ZIKV**	Increased STAT3 (pY705) phosphorylation	In vitro ZIKV infection	FSS13025	Primary Müller cells (mouse)	[92]
	Increased STAT3 pathway activity	In vivo ZIKV infection	Brazil-ZKV2015, PRVABC59	PBMCs (rhesus monkeys)	[93]
**MuV**	STAT3 protein degradation	In vitro viral protein expression (MuV V) and MuV infection	Enders strain	Human fibrosarcoma-derived cell line (2fTGH)	[94]
**MeV**	Reduced STAT3 transcriptional activity	In vitro viral protein expression (MeV V)	Edmonston strain-derived sequence	Human fibrosarcoma-derived cell line (2fTGH)	[95]
**IAV**	Decreased STAT3 (pY705) phosphorylation	In vitro IAV infection	H1N1/54, H5N1/483	Alveolar epithelial cells	[96]
	Increased STAT3-dependent transcription (ANGPTL4)	In vivo IAV infection	H1N1 A/PR/8/34	BALB/c mice	[103]
**HEV**	p-STAT3 impaired nuclear translocation	In vitro viral protein expression (ORF3)	Hyderabad strain-derived sequence (genotype 1)	Human hepatoma cell line (Huh7)	[98]
**RABV**	p-STAT3 impaired nuclear translocation	In vitro viral protein expression (RABV P)	CVS strain-derived sequence	Fibroblast-derived cell line (COS-7)	[99]
**hMPV**	Decreased STAT3 (pY705) phosphorylation and nuclear translocation	In vitro hMPV infection	CAN97-83	Lung adenocarcinoma cell line (A549)	[100]

**Abbreviations:** ANGPTL4, angiopoietin-like protein 4; CVS, challenge virus standard; DCs, dendritic cells; EBNA2, Epstein–Barr virus nuclear antigen 2; EBV, Epstein–Barr virus; HBV, hepatitis B virus; HBx, hepatitis B virus X protein; HCC, hepatocellular carcinoma; HCMV, human cytomegalovirus; HCV, hepatitis C virus; HELFs, human embryonic lung fibroblasts; HEV, hepatitis E virus; hMPV, human metapneumovirus; HSAECs, human small airway epithelial cells; HSCs, hepatic stellate cells; HUVECs, human umbilical vein endothelial cells; IAV, influenza A virus; IE1, intermediate-early protein 1; IE2, intermediate-early protein 2; JFH-1, Japanese fulminant hepatitis; KSHV, Kaposi’s sarcoma-associated herpesvirus; LMP1, latent membrane protein 1; MEFs, mouse embryonic fibroblasts; MeV, measles virus; MeV V, measles virus viral protein V; miRNA, microRNA; MuV, mumps virus; MuV V, mumps virus viral protein V; NPC, nasopharyngeal carcinoma; NS5A, non-structural protein 5A; NSs, non-structural proteins; PBMCs, peripheral blood mononuclear cells; PHHs, primary human hepatocytes; RABV, rabies virus; RVFV, Rift Valley fever virus; STAT3, signal transducer and activator of transcription 3; Tg, transgenic; u-STAT3, unphosphorylated STAT3; vIL-10, viral IL-10; vIL-6, viral IL-6; VZV, varicella-zoster virus; ZIKV, Zika virus.

### Viral suppression of STAT3 function

In the acute phase, viral suppression of STAT3 reduces the host cell's ability to respond to inflammatory cytokines. On the other hand, inhibiting STAT3 also removes negative feedback on the antiviral response. To understand the beneficial effect of blocking STAT3 for viruses, it thus requires a temporal dissection of each individual virus/STAT3 interaction. Most viruses that suppress STAT3, however, do this to avoid the antiviral pressure exerted by STAT3 responsive genes in the acute phase of infection (Fig 2B, Table 1). Mumps virus (MuV) viral protein V (MuV V) induces STAT3 degradation by promoting STAT3-directed ubiquitin E3 ligase complexes [94]. Similarly, measles virus (MeV) viral protein V (MeV V) reduces STAT3-mediated transcription but through an unknown mechanism that is, however, independent of ubiquitin ligase subunits [95]. Influenza A virus (IAV) infection induces STAT3 activation in the early phase of the inflammatory response. As the infection progresses, STAT3 activity is suppressed to a degree that inversely correlates with the pathogenicity of each IAV strain. For instance, the highly pathogenic avian influenza strain H5N1 impairs pY705 phosphorylation, but in the case of the low pathogenic seasonal H1N1 strain this decrease is even more pronounced [96]. This inhibition could be partly mediated by viral protein NS1, which increases *SOCS3* expression [97]. Other viruses have developed alternative strategies to impair STAT3 function, such as manipulating its subcellular localization during infection. Hepatitis E virus (HEV) ORF3 protein blocks the nuclear translocation of p-STAT3 [98]. Likewise, in rabies virus (RABV) infections, viral protein P associates with p-STAT3 in the cytoplasm, impeding its nuclear translocation. In addition, P protein interferes with gp130 receptor signaling [99]. Human metapneumovirus (hMPV) infection prevents the nuclear translocation of STAT3 in a cytokine-specific manner, as this was only observed following stimulation with IL-6 and not in case of interleukin 22 (IL-22) [100]. Contrary to the occasions where HCMV induces STAT3 phosphorylation [78,79], HCMV can also rapidly disrupt IL-6/STAT3 signaling in U-373 cells by sequestering u-STAT3 to the nucleus via viral protein IE1 [101]. Apart from inducing STAT3 activation, KSHV can also target and inhibit STAT3 or its activators in vitro through a panel of virally encoded miRNAs. KSHV miR-K6-5, miR-K8, and miR-K9* reduce STAT3 levels, while upon IL-6 treatment, miR-K6-5 and miR-K9 decrease PKCδ and interleukin 1 receptor-associated kinase 1 (IRAK1) expression, respectively, which is accompanied by reduced p-STAT3 levels [102]. Whether in the end KSHV-induced STAT3 activation or the negative regulation of STAT3 by viral miRNAs act predominantly in endothelial cells remains unclear. But it is conceivable that both opposing mechanisms are required in a time-dependent manner to regulate the transition from the latent to the lytic stage of the viral life cycle.

## Consequences of viral perturbations in STAT3 activity

### Recalibration of apoptosis dynamics

**Fig 3:**
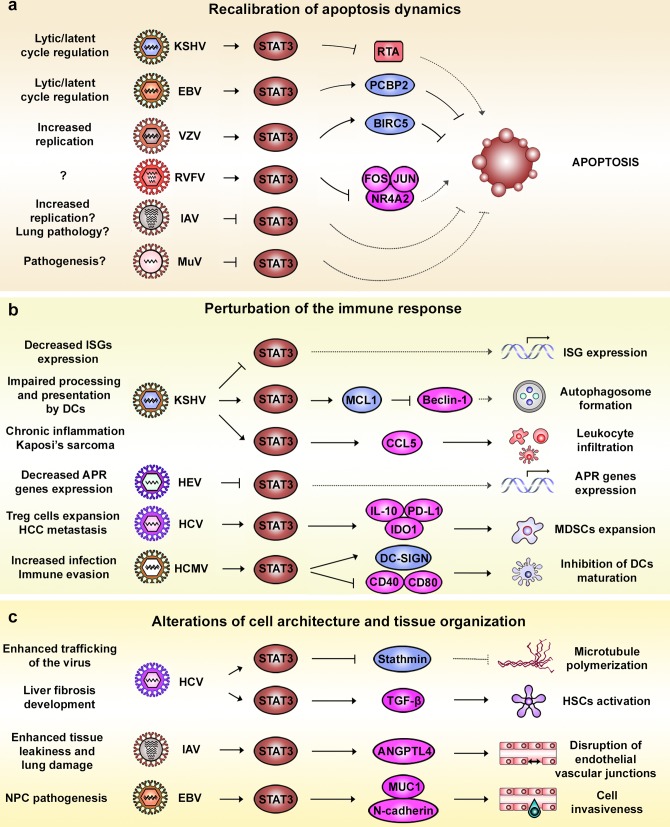
Viral replicative advantages and pathological consequences related to STAT3-altered function. **(A)** Virus-induced perturbation of STAT3 as regulator of apoptosis. In the context of viral infections, apoptosis can be restrained via STAT3, since it favors the expression of antiapoptotic factors (e.g., *PCBP2* and *BIRC5*) or prevents proapoptotic ones (e.g., RTA, *FOS*, *JUN*, and *NR4A2*). In contrast, inhibition of STAT3 by viruses such as IAV and MuV has been associated with the induction of the apoptotic process. **(B)** Viral manipulation of STAT3 and its effect on immune responses. Viral inhibition of STAT3 can induce a decrease of ISG and APR gene expression and favor immune evasion, as in the case of KSHV and HEV. Virus-mediated STAT3 activation can also have immunosuppressive actions such as impairing DC function (KSHV and HCMV) and favoring the expansion of MDSCs (HCV). In other cases, the proinflammatory actions of STAT3 have been associated with the development of host pathologies such as cancer (KSHV). **(C)** Virus-induced alteration of STAT3 and its impact on cell and tissue organization. STAT3 activation during HCV infection has been associated with alterations of the MT network. This represents a potential advantage for HCV by favoring virus trafficking along MTs. At the tissue and organ level, STAT3 activation has been associated with the development of fibrosis (HCV), the disruption of endothelial vascular junctions (IAV), and enhanced cell invasion, which favors cancer development (EBV). ANGPTL4, angiopoietin-like protein 4; APR, acute phase response; BIRC5, baculoviral IAP repeat-containing protein 5; CCL5, C-C motif chemokine ligand 5; DCs, dendritic cells; DC-SIGN, dendritic cell-specific ICAM-3-grabbing non-integrin 1; EBV, Epstein–Barr virus; FOS, proto-oncogene c-Fos; HCC, hepatocellular carcinoma; HCMV, human cytomegalovirus; HCV, hepatitis C virus; HEV, hepatitis E virus; HSCs, hepatic stellate cells; IAV, influenza A virus; IDO1, indoleamine 2,3-dioxygenase 1; IL-10, interleukin 10; ISG, interferon-stimulated gene; JUN, proto-oncogene c-Jun; KSHV, Kaposi’s sarcoma-associated herpesvirus; MCL1, induced myeloid leukemia cell differentiation protein Mcl-1; MDSCs, myeloid-derived suppressor cells; MT, microtubule; MUC1, mucin 1 cell surface associated; MuV, mumps virus; NPC, nasopharyngeal carcinoma; NR4A2, nuclear receptor subfamily 4 group A member 2; PCBP2, poly(rC)-binding protein 2; PD-L1, programmed cell death 1 ligand 1; RTA, R transactivator; RVFV, Rift Valley fever virus; STAT3, signal transducer and activator of transcription 3; TGF-β, transforming growth factor beta; T_reg_, regulatory CD4^+^ T cell; VZV, varicella-zoster virus.

Apoptosis is perhaps the most primordial response of a host cell to infection, designed to thwart the virus spread. Generally, viruses need to prevent host cell apoptosis to maintain a compartment of infected cells [104]. However, there are also examples where viruses induce apoptosis to spark the release of virions and galvanize viral spread [105]. STAT3 is mainly considered a negative regulator of apoptosis by up-regulating the expression of several antiapoptotic factors [106] (Fig 3A). IAV H5N1 causes higher pY705 levels than seasonal H1N1. Therefore, apoptosis is delayed during H5N1 infection, allocating additional time to infected cells for progeny virus production. Ultimately, this leads to an accumulation of apoptotic cells at later stages [96]. Similarly, VZV prevents apoptosis by increasing STAT3 phosphorylation, which up-regulates baculoviral IAP repeat-containing protein 5 (*BIRC5*) expression, a VZV host factor belonging to the family of inhibitors of apoptosis (IAP) [90]. During EBV infection, virus-induced STAT3 activation up-regulates poly(rC)-binding protein 2 (*PCBP2*) expression, limiting susceptibility of latently infected cells to lytic signals and fostering persistence [107]. This goes as well for KSHV, in which STAT3 restrains the exit from latency into the lytic cycle by repressing the expression of the viral protein R transactivator (RTA) [108]. MuV is yet another example in which the cytopathic effects of infection are associated with the induction of apoptosis, partly via V protein-mediated STAT3 degradation [94]. Finally, RVFV reins in apoptosis by enhancing the nuclear translocation of phosphorylated STAT3 and impairs the expression of proapoptotic genes such as proto-oncogene c-Fos (*FOS*), proto-oncogene c-Jun (*JUN*), and nuclear receptor subfamily 4 group A member 2 (*NR4A2*) [77].

### Perturbing the immune response

The benefit for a virus to dampen STAT3 signaling lies in controlling antiviral innate immunity responses such as the APR (Fig 3B). Many of the APR genes are modulators of inflammation. C-reactive protein (CRP) for example is a target gene of STAT3 and has several biological functions related to nonspecific host defense [109]. Increased plasma levels of metal-binding APRs (e.g., haptoglobin and hemopexin) help protect host cells from iron loss during infection and the associated injury. Moreover, they act as scavengers for potentially damaging free oxygen radicals. Protease inhibitors among APR genes (e.g., alpha-1-antitrypsin) neutralize lysosomal proteases. These inhibiting factors are released in response to tissue infiltration of activated neutrophils and macrophages, modulating the activity of proinflammatory enzyme cascades. HEV impairs the expression of these APR genes by inhibiting STAT3, attenuating inflammatory responses and creating a favorable environment for viral replication and survival [98].

In contrast to HEV, the KSHV-mediated activation of STAT3 is associated with increased expression of C-C motif chemokine ligand 5 (CCL5) [19], a potent chemoattractant for monocytic cells, eosinophils, NKs, and DCs [110]. Many of these cell types have been shown to be present in Kaposi’s sarcoma lesions, suggesting that STAT3 contributes to the chronic inflammatory state observed in this pathology [19]. Moreover, KSHV-induced STAT3 activation correlates with up-regulated induced myeloid leukemia cell differentiation protein Mcl-1 (MCL1) expression levels, which can be reverted by inhibiting STAT3 [89]. MCL1 inhibits Beclin-1, a positive regulator of autophagosome formation, to interfere with antigen processing and presentation by DCs to avoid recognition and clearance [89]. KSHV also inhibits STAT3 via the action of viral miRNAs, and by doing so it hinders the expression of ISGs such as *CXCL10*, *ISG15*, *IFITM1*, *IRF1*, *OAS2*, and *MX1* [102]. The vIL-10 coded by HCMV up-regulates expression of its receptor DC-SIGN in DCs, their target cells [81,111]. vIL-10 stimulation of DCs also prevents the expression of costimulatory molecules (i.e., CD40, CD80, and CD86), inhibiting maturation of DCs, enhancing their susceptibility to infection, and hampering the immune response [81]. Chronic HCV infection has been associated with the presence of myeloid-derived suppressor cells (MDSCs), a heterogeneous population of myeloid cells that suppress the function of NK, CD4^+^, and CD8^+^ T cells [112]. Analysis of myeloid and lymphoid cells from chronically HCV-infected patients has shown that activation of STAT3 up-regulates the expression of suppressive genes (i.e., IL-10, programmed cell death 1 ligand 1 [PD-L1], indoleamine 2,3-dioxygenase 1 [IDO1]) in monocytic cells. They acquire MDSC-like characteristics and favor the expansion of T_reg_ cells [113,114]. MDSCs have been linked to an increased tumor burden and a higher metastasis rate in patients with HCC and in liver cancer mouse models [115]. Thus, by the STAT3-mediated induction of MDSCs, HCV can establish a microenvironment that supports viral immune evasion and accelerates HCC development.

### Altering cell architecture and tissue organization

STAT3 also plays a role in cell morphology, which viruses exploit to promote viral persistence, with grave consequences for host cell physiology (Fig 3C). HCV-induced p-STAT3 directly controls microtubule (MT) dynamics through contact inhibition with stathmin [74]. Both HCV core and NS5A are transported along MTs [116]. Moreover, HCV core integrates into the MT lattice by a direct binding to tubulin [117]. Viral attenuation of stathmin enhances intracellular trafficking of the virus and increases replication [74]. In addition, regenerative STAT3 activation in HSCs precipitates fibrotic gene expression (i.e., *TGF-β1*, *TIMP-1*) [28], eventually leading to cirrhosis, which constitutes the procarcinogenic field on which most HCCs grow [118]. IAV triggers a STAT3-mediated up-regulation of angiopoietin-like protein 4 (ANGPTL4), a protein that compromises the integrity of endothelial vascular junctions. This leads to enhanced tissue leakiness and exacerbation of inflammatory lung damage in infected mice [103]. EBV is the most distinct etiological agent for the development of nasopharyngeal carcinoma (NPC), a type of cancer in which STAT3 activation or overexpression is associated with more than 75% of tumors in regions where EBV is endemic [119]. EBV-mediated activation of STAT3 spurs cell invasiveness in vitro, and constitutive expression of STAT3 in NPC cell lines results in an increase of mesenchymal markers such as fibronectin and N-cadherin [120]. In accordance, STAT3 activation via LMP1 induces the expression of mucin 1 cell surface-associated (MUC1), a glycoprotein involved in the early steps of cancer cell detachment [121].

## Disruption of STAT3 function as antiviral therapy

In the cases where STAT3 activity has a proviral or pathogenic effect, blocking STAT3 represents an interesting therapeutic strategy. Unfortunately, no molecule directly targeting STAT3 has received Food and Drug Administration (FDA) approval for any pathology so far [122], and candidate compounds targeting viral disease have not advanced beyond preclinical evaluation (Table 2). Small-molecule inhibitors targeting STAT3 phosphorylation (e.g., Cpd188, IB-32, Stattic) or dimerization (e.g., STA-21, S3I-201) have been evaluated as antivirals in vitro or in animal models. For instance, HCV replication but not entry is inhibited by STA-21, S3I-201, Cpd188, and IB-32 in Huh7 hepatoma cells or derivatives thereof [58,74,123]. Similarly, S3I-201 and Stattic reduce HCMV replication in cell culture [101], while S3I-201 limits VZV infection both in vitro and in animal models [90]. Oligodeoxynucleotide decoys (ODNs) are DNA-binding domain inhibitors that compete for binding of transcription factors with endogenous promoter sequences in their target genes. STAT3-targeting ODNs significantly decrease HBV RNA expression and DNA replication in hepatoma cell lines [124].

**Table 2:** STAT3 signaling inhibitors, their mechanisms and in vitro antiviral applications.

Molecule	Targets	Molecule class	Mechanism of action	Antiviral effect	Refs
Cpd188	STAT3	Non-peptide small molecule	Inhibition of STAT3 phosphorylation	HCV	[58]
IB-32	STAT3	Non-peptide small molecule	Inhibition of STAT3 phosphorylation	HCV	[123]
STA-21	STAT3	Non-peptide small molecule	Inhibition of STAT3 dimerization	HCV	[74]
S3I-201	STAT3	Non-peptide small molecule	Inhibition of STAT3 dimerization	HCV VZV HCMV	[74] [90] [101]
Stattic	STAT3	Non-peptide small molecule	Inhibition of STAT3 phosphorylation	HCMV	[101]
Sorafenib	VEGFR PDFGR BRAF JAK2 STAT3	Tyrosine kinase inhibitor	Inhibition of STAT3 phosphorylation	HCMV*	[130]
Resveratrol	JAK1 STAT3	Natural product	Inhibition of STAT3 phosphorylation	VZV* EBV	[91] [126,127]
Curcumin	JAK1 JAK2 JAK3 STAT3	Natural product	Inhibition of STAT3 nuclear localization	HCMV	[101]
Oligodeoxynucleotide decoy	STAT3	DNA-binding modifier	Inhibition of STAT3 transcriptional activity	HBV	[124]

*Antiviral effect via STAT3 not determined.

**Abbreviations:** BRAF, serine/threonine-protein kinase B-raf; EBV, Epstein–Barr virus; HBV, hepatitis B virus; HCMV, human cytomegalovirus; HCV, hepatitis C virus; JAK1, Janus kinase 1; JAK2, Janus kinase 2; JAK3, Janus kinase 3; PDFGR, platelet-derived growth factor receptor; STAT3, signal transducer and activator of transcription 3; VEGFR, vascular endothelial growth factor receptor; VZV, varicella-zoster virus.

In addition, several natural products such as resveratrol or curcumin have been described to exhibit STAT3 inhibitory properties [125]. Resveratrol impairs EBV and VZV infection. For EBV, at least, it has been demonstrated that resveratrol suppresses STAT3 phosphorylation [126,127], while the antiviral mechanism by which resveratrol inhibits VZV is not yet understood [91]. Curcumin hinders HCMV replication in U373 cells by reducing nuclear accumulation of STAT3 [101], and while it exerts antiviral properties for IAV [128] and HCV [129], a mechanistic link to STAT3 has not been demonstrated yet.

The multikinase inhibitor sorafenib exhibits an antiviral effect against various HCMV strains by inhibiting the expression of immediate early genes of HCMV at clinically relevant concentrations [130]. However, sorafenib is not selective for STAT3; therefore, it is likely that a combination of unspecific effects may account for the observed antiviral effect of sorafenib on HCMV.

## Outlook

STAT3 is a key regulator in inflammation and tissue regeneration triggered by almost every pathogenic infection. Therefore, viruses must deal with STAT3 activity by either curtailing it or employing it. STAT3 dependencies of viruses put a spotlight on the diverse role of signal transduction during viral infections and represent a target for potential antiviral strategies. Deregulated STAT3 signaling is an oncogenic driver and is associated with virus-induced complications, including cancers. However, targeting STAT3 during viral infection and cancer is currently an untapped reservoir, and the question still remains as to why it has not yet resulted in a broad range of clinical applications.

Currently, unspecific tyrosine kinase inhibitors (e.g., sorafenib) and monoclonal antibodies (e.g., tocilizumab) that block upstream components in the STAT3 pathway are readily administered to patients as cancer chemotherapeutics [131,132]. Similarly, other indirect STAT3-targeting strategies, including the modulation of STAT3 regulators, are promising. These include the use of histone deacetylase or proteasome inhibitors that promote expression of the endogenous STAT3 inhibitors SOCS3 and PIAS3, respectively [133]. While the use of approved indirect STAT3 modulators in clinical practice allows an indirect safety evaluation for STAT3-targeting strategies, their use does not allow conclusions on the specific clinical tolerance and efficacy of a STAT3-based antiviral approach.

Several natural products targeting STAT3 are currently being explored and seem promising; however, many (including curcumin and resveratrol) have been described as pan-assay interference compounds (PAINs). In other words, it currently cannot be ruled out that the observed effects of these natural compounds are due to an interference with the experimental readout rather than an interaction with their specific targets [134].

Due to multiple and redundant pathways that converge in STAT3 activation, direct STAT3-targeting agents would be a gold standard to assess the potential benefit of this approach. One reason why we have not observed a breakthrough in STAT3-targeting drugs so far may be that transcription factors are notoriously difficult to target and that many of the STAT3 inhibitors evaluated to date have shown to be problematic regarding their potency, bioavailability, and specificity [122]. Nevertheless, as we have explored in this review, there is strong scientific rationale to continue the development of novel STAT3-targeting therapies. Recently emerged agents that appear encouraging include AZD9150, an antisense oligonucleotide targeting *STAT3* mRNA that is in early phase I and II studies for advanced solid and hematological cancers [135–137], and napabucasin, a small-molecule inhibitor that has advanced to phase III clinical trials [138]. The evaluation of these and similar compounds for the treatment of cancers is expected to result in a broad range of clinical applications and holds great promise for future antiviral strategies as well.
